# Auxin (Indole-3-acetic acid) modulation of quorum sensing enhances phage susceptibility in *Klebsiella pneumoniae*

**DOI:** 10.1007/s10096-026-05454-z

**Published:** 2026-03-27

**Authors:** Antonio Barrio-Pujante, Lucia Blasco, Inés Bleriot, Laura Fernández-García, Clara Ibarguren-Quiles, Lucia Arman, Belén Aracil, Luis Eduardo López-Cortes, Olaya Menéndez-Rodríguez, Patricia Fernández-Grela, María Pérez Vázquez, Maria Tomás

**Affiliations:** 1 Multidisciplinary and Translational Microbiology group (MicroTM), Biomedical Research Institute of A Coruña (INIBIC), Microbiology Service, University Hospital of A Coruña (CHUAC), University of A Coruña (UDC), A Coruña, Spain; 2 Laboratorio de Referencia e Investigación de Resistencias Antibióticas e Infecciones Sanitarias, Centro Nacional de Microbiología, Instituto de Salud Carlos III, Majadahonda, Madrid Spain; 3 Unidad Clínica de Enfermedades Infecciosas y Microbiología, Hospital Universitario Virgen Macarena, Seville, Spain; 4 Departamento de Medicina y Microbiología, Facultad de Medicina, Universidad de Sevilla, Seville, Spain; 5 Microbiology Service Hospital University Puerta de Hierro, Madrid, Spain; 6 CIBER de Enfermedades Infecciosas (CIBERINFEC), Instituto de Salud Carlos III, Madrid, Spain; 7 Proyecto de Medicina de Precisión contra las resistencias Antimicrobianas, MEPRAM, Madrid, Spain

**Keywords:** Indole-3-acetic acid, Auxin, Quorum sensing, SdiA, Phage therapy, Phage defense mechanisms, Anti-phage defense systems, *Klebsiella pneumoniae*

## Abstract

**Purpose:**

*Klebsiella pneumoniae* is a multidrug-resistant (MDR) bacterium that has emerged as a major global public health threat. The decreasing effectiveness of conventional antibiotics has renewed interest in bacteriophage therapy as a promising alternative antibacterial strategy. However, the rapid emergence of phage resistance remains a critical limitation. This study aimed to investigate whether indole-3-acetic acid (IAA) can modulate bacterial quorum sensing (QS) and enhance phage therapy by modulating the QS regulator SdiA.

**Methods:**

Experiments were performed using the lytic bacteriophage VAC7 and a clinical *K. pneumoniae* ST16-OXA48 strain. The minimum inhibitory concentration (MIC) of IAA was determined, and sub-inhibitory concentrations were used to assess QS gene expression by RT-qPCR, phage infection dynamics, and bacterial proteomic responses.

**Results:**

RT-qPCR analysis demonstrated that IAA significantly reduced the expression of the QS regulator gene *sdiA* while increasing *luxS* expression. Phage infection assays showed enhanced bactericidal activity of VAC7 against the ST16-OXA48 clinical isolate in the presence of IAA. Proteomic analysis revealed a reduced abundance of several bacterial phage defense proteins in the presence of IAA, including GmrSD restriction endonuclease, bacteriophage control infection (BCI), mRNA interferase PemK, and abortive infection protein. Proteins associated with phage receptors, such as LamB, OmpK36, OmpC, and FhuA, were detected under all conditions but reduced when the phage is present. The detection of multiple phage structural, functional and anti-phage defense system proteins was consistent with active phage replication.

**Conclusions:**

IAA, a plant-derived auxin, modulates bacterial QS and SdiA, and reduces phage resistance mechanisms in *K. pneumoniae*. These findings highlight the potential of IAA, and possibly other auxins, as adjuvants in phage therapy and as versatile components of combination antimicrobial strategies.

**Supplementary Information:**

The online version contains supplementary material available at 10.1007/s10096-026-05454-z.

## Introduction

*Klebsiella pneumoniae* is a Gram-negative enterobacterium widely distributed in the environment and commonly found on mucosal surfaces of animals and humans. It is an opportunistic pathogen that has evolved into a major clinical and public health threat worldwide due to its remarkable capacity to acquire and disseminate antimicrobial resistance (AMR) genes [1–3]. In particular, multidrug-resistant (MDR) and carbapenemase-producing *K. pneumoniae* strains have become prevalent in healthcare settings, severely limiting available therapeutic options.

In this context of increasing clinical urgency, bacteriophage (phage) therapy has emerged as a promising alternative or complementary approach to conventional antibiotics. Phages are natural bacterial viruses that specifically infect and lyse bacteria offering several advantages, including high host specificity, low toxicity, synergy with antibiotics, and relatively low production costs [4–6]. Despite these benefits, the rapid emergence of bacterial resistance to phages remains a major obstacle to the widespread clinical implementation of phage therapy.

Bacteria have evolved a diverse array of defense mechanisms to evade phage infection. These include inhibition of phage adsorption through biofilm formation or receptor modification [7, 8]; prevention of phage DNA injection via superinfection exclusion (Sie) systems [9]; degradation of viral nucleic acids by restriction–modification (R–M) systems or CRISPR–Cas immunity [10, 11]; inhibition of phage DNA replication through systems such as bacteriophage exclusion (BREX) [12]; and abortive infection mechanisms, including toxin–antitoxin (TA) and CBASS systems, in which infected cells undergo programmed death to prevent phage propagation [9, 13]. In addition, quorum sensing (QS) has recently been identified as a regulatory layer influencing bacterial defense mechanisms against phages [14].

QS is a cell-density-dependent communication system that enables bacteria to coordinate gene expression through the production and detection of signaling molecules known as autoinducers (AIs). In *K. pneumoniae*, QS primarily relies on the autoinducer-2 (AI-2) system, a conserved interspecies signaling pathway mediated by the LuxS synthase. In parallel, *K. pneumoniae* encodes SdiA, an orphan LuxR-type QS regulator that detects acyl-homoserine lactones (AHLs) produced by other bacterial species [15, 16]. SdiA plays a key role in the regulation of virulence-associated traits, including biofilm formation, fimbrial expression, and the production of QS signaling molecules [15]. Importantly, previous studies have demonstrated that inhibition of SdiA enhances susceptibility to phage infection in *K. pneumoniae* [17].

Indole-3-acetic acid (IAA) is a major plant auxin that regulates plant growth, development, and responses to biotic and abiotic stress. Beyond its role in plants, IAA functions as an important bacterial signaling molecule, influencing primary and secondary metabolism, biofilm formation, virulence factor production, auxin catabolism, and host colonization [18–20]. Several bacterial biosynthetic pathways for IAA have been characterized, most notably the indole-3-acetamide (IAM) and indole-3-pyruvate (IPyA) pathways, both of which utilize tryptophan as a precursor [21].

Recent studies have shown that IAA modulates central metabolic pathways in *Escherichia coli* [22] and regulates bacterial physiology, gene expression, and phage susceptibility in *Serratia plymuthica*, including enhanced adsorption of capsule-dependent phages [20]. Additionally, endogenous indole has been reported to inhibit SdiA activity in enterobacteria [23], these findings raise the possibility that indole-derived compounds may interact with QS-related pathways. However, in Enterobacteriaceae, the role of IAA in modulating QS-regulated processes remains to be fully elucidated.

In this study, we investigated the role of exogenously supplied IAA as a natural QS-modulating compound capable of enhancing phage infection in *K. pneumoniae*. Using sub-inhibitory concentrations of IAA, we evaluated its impact on SdiA regulation, QS signaling, bacterial defense mechanisms, and susceptibility to lytic phage infection. Our findings provide new insights into the potential of auxins as adjuvants to improve phage therapy efficacy against MDR *K. pneumoniae*.

## Materials and methods

### Bacterial strain and bacteriophage

The lytic bacteriophage vB_KpnM_VAC7 (hereafter referred to as VAC7), belonging to the family Drexlerviridae and genus Webervirus (GenBank accession MZ428225.1; BioProject PRJNA739095), was used in this study. The host strain was a clinical *Klebsiella pneumoniae* ST16-OXA48 isolate (GenBank accession WRXF00000000), which was previously shown to be susceptible to phage VAC7 [24]. The bacterial strain was obtained from the Spanish National Centre for Microbiology (Madrid, Spain).

### Phage propagation and purification

Phage VAC7 was propagated using the double-layer agar method as previously described [24]. Briefly, an overnight culture of *K. pneumoniae* was diluted 1:100 in Luria–Bertani (LB) broth (1% tryptone, 0.5% yeast extract, 1% NaCl) and incubated at 37 °C with shaking until reaching an optical density at 600 nm (OD₆₀₀) of 0.5. Subsequently, 50 µL of phage suspension was mixed with 200 µL of bacterial culture and combined with 4 mL of molten top agar (0.5% NaCl, 1% tryptone, 0.4% agar). The mixture was poured onto tryptone agar plates (0.5% NaCl, 1% tryptone, 1.5% agar) and incubated at 37 °C for 24 h.

Plates were then flooded with SM buffer (0.1 M NaCl, 10 mM MgSO₄, 20 mM Tris-HCl, pH 7.5) and gently agitated at room temperature for 3 h. The resulting phage suspension was collected, treated with 1% chloroform for 20 min to lyse residual bacterial cells, and centrifuged at 3,400 × g for 15 min. The supernatant was filtered through sterile 0.45 μm PES syringe filters (Branchia) and stored at 4 °C until use.

### Determination of the minimum inhibitory concentration of IAA

The minimum inhibitory concentration (MIC) of indole-3-acetic acid (IAA; Sigma-Aldrich) against *K. pneumoniae* ST16-OXA48 was determined using a broth microdilution assay. Nine two-fold serial dilutions of IAA were prepared in Mueller–Hinton broth (MHB) in 96-well microtiter plates. Each well was inoculated with bacterial cells to a final concentration of 5 × 10⁵ CFU/mL. Wells containing only MHB served as negative controls, while wells with MHB inoculated with bacteria but without IAA served as positive growth controls.

Plates were incubated at 37 °C for 24 h, and the MIC was defined as the lowest concentration of IAA at which no visible bacterial growth was observed. All assays were performed in triplicate.

### Quantification of quorum sensing gene expression

The effect of sub-inhibitory IAA concentrations on the expression of QS genes was evaluated by RT-qPCR. An overnight culture of *K. pneumoniae* ST16-OXA48 was inoculated (1:100 dilution) into 20 mL LB broth supplemented with 0.5 mM IAA and incubated at 37 °C with shaking at 180 rpm until an OD₆₀₀ of 0.3 was reached. Control cultures were grown under identical conditions without IAA but with the same volume of dimethyl sulfoxide (DMSO) solvent, used to prepared IAA.

Duplicate 1 mL aliquots were collected for RNA extraction using the High Pure RNA Isolation Kit (Roche), following the manufacturer’s instructions. RNA concentration and purity were assessed using a NanoDrop spectrophotometer (NanoDrop Technologies), and samples were adjusted to 50 ng/µL with nuclease-free water.

RT-qPCR was performed using the iTaq™ Universal SYBR^®^ Green One-Step Kit (Bio-Rad) on a LightCycler^®^ 480 system. Gene-specific primers targeting *sdiA*, *luxS*, and the reference gene *rho* were used, gene *rho* was previously validated like reference gene in *K. pneumoniae* using the BestKeeper algorithm, showing high expression stability (*r* = 0.816, *p* = 0.001) [25] (Primers used in Table 1). Relative gene expression levels were calculated using the comparative Ct method. Statistical significance was determined using Student’s t-test (GraphPad Prism 9.0.0), with *p* < 0.05 considered significant. RT-qPCR experiments were performed using two independent biological replicates, each analyzed in duplicate technical replicates.

**Table 1:** Primers used in this work for *rho*, *sdiA* and *luxS* genes in the RT-qPCR

PRIMER NAME	SEQUENCE	STUDY
Kp_sdiA_Fow	CGATATTGTCGCCCGCTTTG	***sdiA*** **gene (QS regulator)**
Kp_sdiA_Rev	CTGTCTTCCAGGCCGTTTCT	
Kp_rho_Fow	CGACGGCGTACTGGAGATAC	***rho*** **gene (Housekeeping)**
Kp_rho_Rev	TTGGCTGGGGGATACGTAGA	
Kp_luxS_Fow	AGTGCGGGACTTACACCATG	***luxS*** **gene (AI-2 biosynthesis)**
Kp_luxS_Rev	CCAGTTCGTCGTTGCTGTTG	

### Phage infection assays

Phage infection dynamics were assessed by constructing infection curves. An overnight bacterial culture was diluted 1:100 in LB broth and adjusted to an initial OD₆₀₀ of 0.3. Four experimental conditions were established: (i) bacterial growth control, (ii) IAA control (0.5 mM), (iii) phage infection control (MOI = 0.1), and (iv) phage infection (MOI = 0.1) in the presence of 0.5 mM IAA. Each condition was prepared in a final volume of 200 µL in 96-well microplates.

Plates were incubated at 37 °C with shaking at 559 cycles per minute in a BioTek Epoch 2.0 microplate reader (Agilent) for 24 h. Samples from three replicate per condition were collected at 0 h, 3 h, and 24 h post-infection.

Bacterial counts were determined as CFU/mL by serial dilution and plating on LB agar. Phage titers were quantified as PFU/mL using the double-layer agar method [24]. All experiments were conducted in triplicate. Statistical significance at each time point was assessed using Student’s t-test (*p* < 0.05) with GraphPad Prism version 9.0.0.

### Proteomic analysis

Proteomic analyses were performed to assess the impact of IAA on bacterial protein expression during phage infection. Overnight cultures of *K. pneumoniae* ST16-OXA48 were diluted 1:100 in LB broth and incubated at 37 °C until reaching an OD₆₀₀ of 0.3 (approximately 10⁷ CFU/mL). Two experimental conditions were analysed: IAA alone (0.5 mM) and IAA combined with phage VAC7 (MOI = 0.1).

After 3 h of incubation, 25 mL of culture from each condition was harvested, placed on ice for 10 min, and centrifuged at 4,500 × g for 20 min at 4 °C. Cell pellets were frozen at − 80 °C. The following day, pellets were resuspended in phosphate-buffered saline (PBS) and lysed by sonication using an ultrasonic processor (UP200S, Hielscher Ultrasonics) at 80% amplitude with a 0.5 s duty cycle for 90 s on ice. Lysates were centrifuged at 4,500 × g for 20 min at 4 °C, and the supernatants were collected for analysis.

Protein samples (200 ng per sample) were subjected to tryptic digestion following reduction and alkylation, desalted using ZipTip columns, and analyzed by NanoUHPLC-Tims-QTOF mass spectrometry using a TimsTOF Pro instrument (Bruker) equipped with a CaptiveSpray nanoESI source and nanoELUTE chromatograph. Peptides were separated on a ReproSil C18 column (150 × 0.075 mm, 1.9 μm, 120 Å) at 50 °C using a linear gradient of 0.1% formic acid in water (A) and 0.1% formic acid in acetonitrile (B) at a flow rate of 0.4 µL/min.

Data were acquired in positive ionization mode using PASEF-MS/MS with CID fragmentation over an m/z range of 100–1,700. Data acquisition and analysis were performed using Compass HyStar 5.1, TimsControl, DataAnalysis (Bruker), and PEAKS Studio (Bioinformatics Solutions). Protein identification was performed against the *K. pneumoniae* reference database. A blank control (PBS) processed in parallel was included to identify and remove potential contaminants. Protein quantification was carried out using label-free quantification (LFQ) based on peptide intensities. PEAKS Studio parameters included a false discovery rate (FDR) threshold of 1% at the protein level and a minimum of two unique peptides per protein. No batch effects were observed, as all samples were processed and analyzed in the same experiment, and intensity normalization was applied to minimize any potential variability.

## Results

### Minimum Inhibitory Concentration of IAA

The minimum inhibitory concentration (MIC) of indole-3-acetic acid (IAA) against the clinical *Klebsiella pneumoniae* ST16-OXA48 isolate was determined using a broth microdilution assay. The MIC was established at 3 mM. Based on this result, a sub-inhibitory concentration of 0.5 mM IAA, dissolved in DMSO, was selected for all subsequent experiments, to ensure the absence of bactericidal effects while allowing the evaluation of regulatory and modulatory responses.

### IAA Modulates the Expression of Quorum-Sensing Genes

**Fig. 1:**
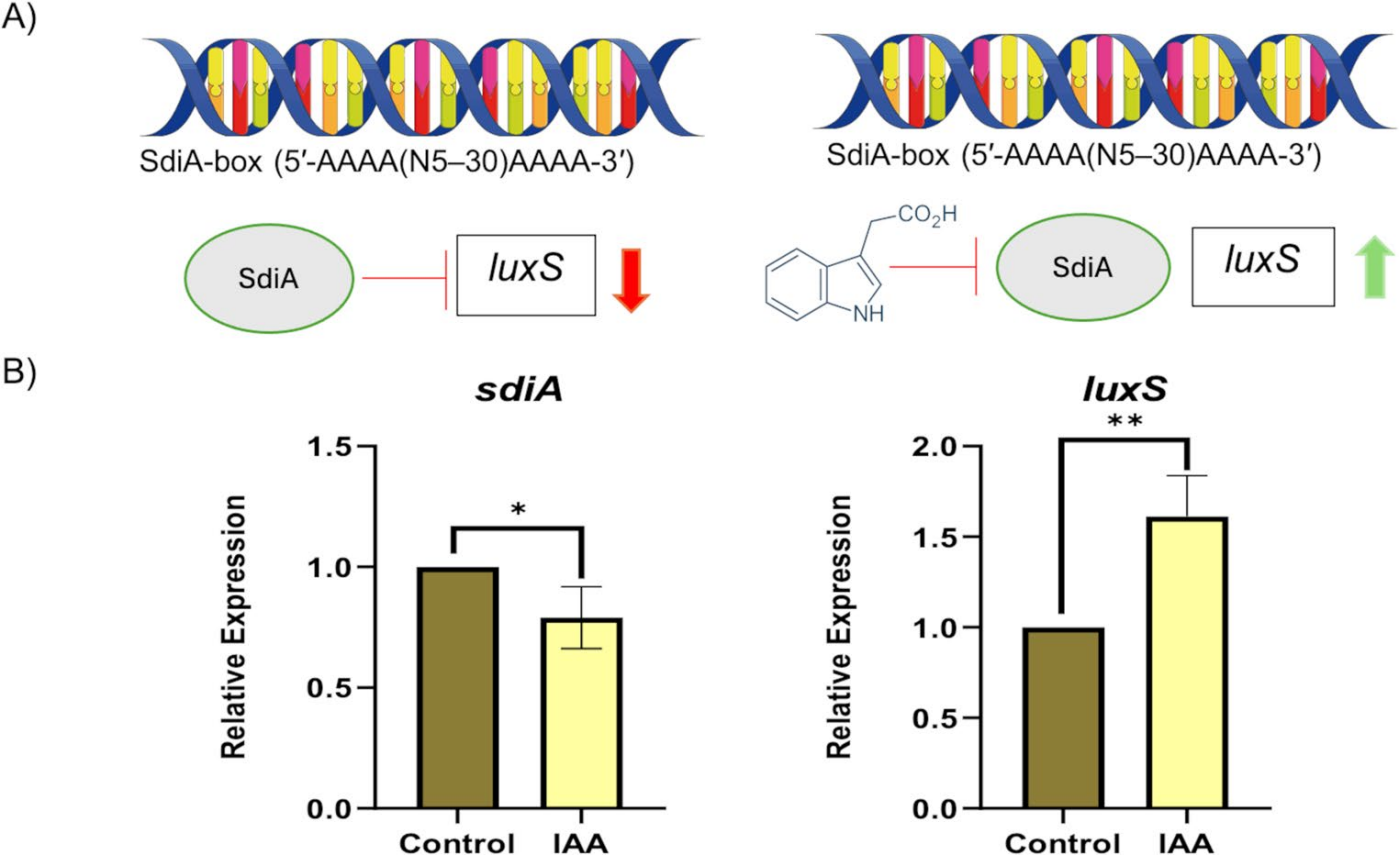
Proposed global action mechanism of exogenous IAA in the bacterial QS cycle. (**A**) The *luxS* gene responsible for AI-2 production has a SdiA binding sequence in its promoter, which repress the transcription of the gene. Exogenous IAA modulates negatively *sdiA* expression, preventing it from binding to the *luxS* promoter and increasing the transcription, thereby activating the AI-2 QS cycle. (**B**) RT-qPCR analysis of the *sdiA* gene and the *luxS* gene by adding exogenous IAA to *K. pneumoniae*, resulting in reduced expression of *sdiA* and increased expression of *luxS*

To evaluate the role of IAA in QS regulation, the expression levels of the *sdiA* and *luxS* genes were quantified by RT-qPCR in the presence of 0.5 mM IAA. As shown in Fig. 1, exposure to IAA resulted in a significant reduction, 0.80 ± 0.02-fold relative to the control (mean ± SEM, *n* = 2) in *sdiA* expression compared with the untreated control. In contrast, *luxS* expression was significantly upregulated, 1.61 ± 0.15-fold relative to the control (mean ± SEM, *n* = 2) under the same conditions.

### IAA Enhances Phage-Mediated Bacterial Killing

The effect of IAA on phage infectivity was assessed through infection curve analyses using phage VAC7 and the *K. pneumoniae* ST16-OXA48 strain. Bacterial growth was monitored under four experimental conditions: untreated control, IAA alone (0.5 mM), phage VAC7 alone (MOI = 0.1, selected to ensure that the effects of IAA could be observed before significant phage-induced lysis occurs), and phage VAC7 in combination with IAA.

**Fig. 2:**
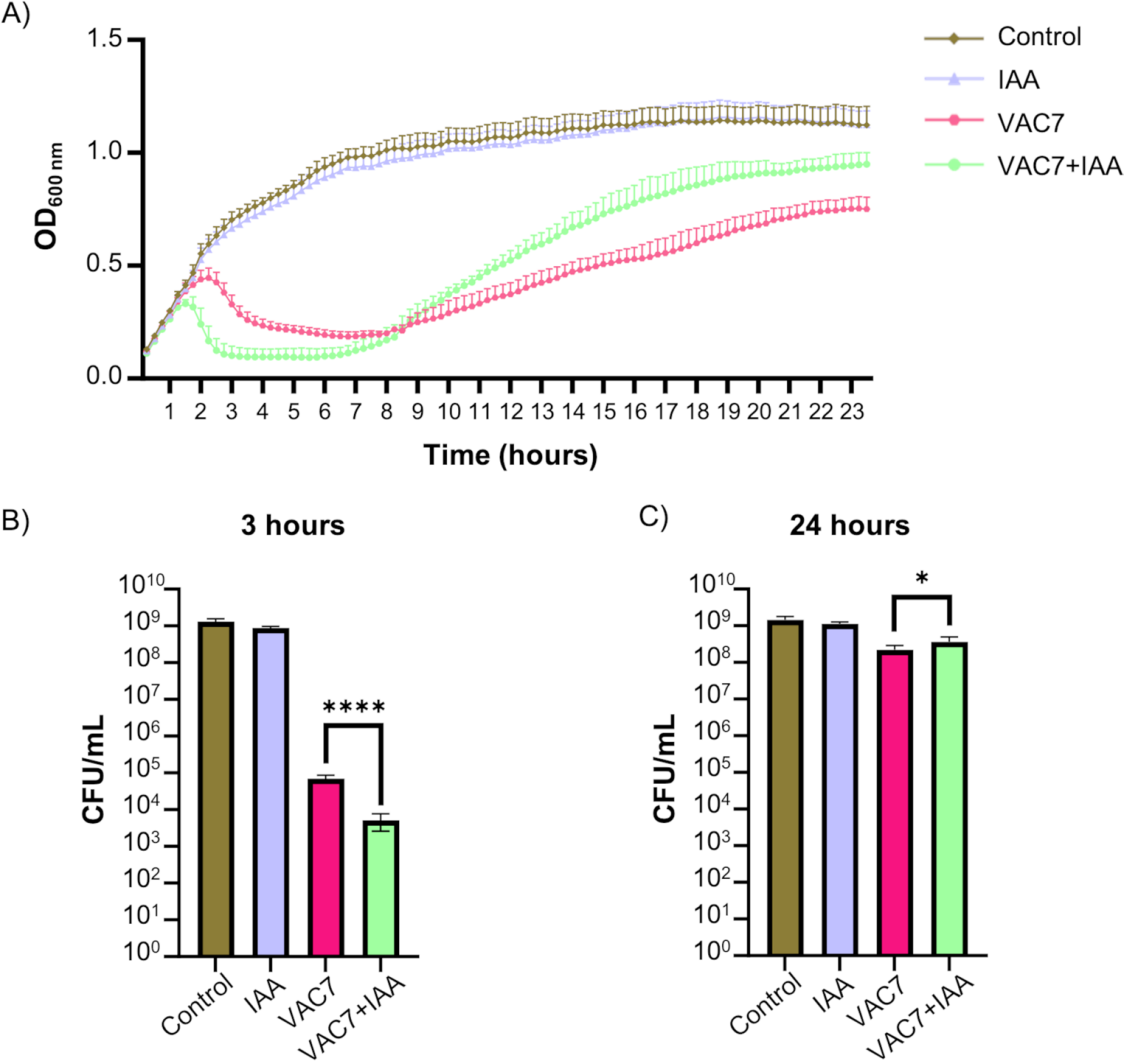
Phage infection assays. (**A**) Infection curve of *K. pneumoniae* ST16-OXA48 with phage VAC7 alone and in combination with IAA. (**B**) CFU/mL of strain ST16-OXA48 infected with phage VAC7 alone and in combination with 0.5 mM IAA at 3 h post-infection. (C) CFU/mL of strain ST16-OXA48 infected with phage VAC7 alone and in combination with 0.5 mM IAA at 24 h post-infection

At 3 h post-infection, cultures treated with the phage-IAA combination exhibited a marked reduction in OD₆₀₀ compared with the phage-only condition, indicating enhanced bacterial killing (Fig. 2A). At 24 h, bacterial growth partially recovered in both phage-treated conditions; however, growth remained lower than in the untreated control.

OD_600_ measurements may be unreliable in lysing cultures, as cell debris can affect absorbance readings, so these observations were corroborated by CFU and PFU quantification. At 3 h post-infection, a statistically significant decrease in CFU/mL was observed in the phage-IAA condition compared with phage alone (Fig. 2B), demonstrating a synergistic effect between IAA and phage VAC7. At 24 h, CFU counts increased, reflecting bacterial regrowth (Fig. 2C). No significant differences in PFU/mL were detected between conditions at any time point (Supplementary Fig. 1S), indicating that IAA did not adversely affect phage replication.

Importantly, no significant differences were observed between the IAA-only condition and the growth control in either OD₆₀₀ measurements (Fig. 2A) or CFU counts (Fig. 2B, C), confirming that IAA at 0.5 mM does not exert detectable toxicity on the bacterial strain.

### Proteomic analysis reveals reduced phage defense in the presence of IAA

To elucidate the molecular mechanisms underlying the enhanced phage activity observed in the presence of IAA, a comparative proteomic analysis was performed using NanoUHPLC-Tims-QTOF mass spectrometry. A total of 3,183 proteins were identified across all samples (Supplementary Table 1S). Of these, 2,269 proteins were detected in the IAA-only condition, whereas 2,775 proteins were identified in the IAA plus phage condition.

Proteins were grouped into three major functional categories: (i) phage defense mechanisms, (ii) QS related proteins, and (iii) phage receptor proteins (Table 2).

**Table 2:** Bacterial proteins of proteomic analysis associated with Phage defense mechanism, QS and Phage Receptors. The table includes Protein name; Accession number (database identifier); − 10LgP value, which represents the statistical confidence of protein identification; Abundance values associated with each condition; Biological function associated with each protein; Bibliographic references and log2-transformed FC values, that represent the ratio of protein abundance in IAA+VAC7 condition relative to the IAA condition. Positive values indicate increased protein abundance, whereas negative values decreased abundance

**PHAGE DEFENSE MECHANISMS**								
**Description**	**Accession no.**		**−10LgP**	**IAA**	**VAC7 + IAA**	**Function**	**Ref.**	**log2FC**
GmrSD restriction endonucleases	A0A919HRM9		1.43E + 08	2.59E + 07	0.00E + 00	Type IV modification-dependent restriction	[39]	−24.62
Bacteriophage control infection (BCI)	A0A0H3GWL4		7.46E + 08	2.08E + 07	0.00E + 00	Infection regulator	[30]	−24.32
mRNA Interferase PemK	A0A2J5A2H8		5.16E + 09	1.23E + 07	0.00E + 00	TA system	[29]	−23.54
Abortive infection Protein	G7RUW5		1.82E + 09	3.43E + 08	4.14E + 06	Abortive system	[40]	−6.35
**QUORUM SENSING**								
**Description**		**Accession no.**	**−10LgP**	**IAA**	**IAA+VAC7**	**Function**	**Ref.**	**log2FC**
Aldolase LsrF		A0A5C2LKW9	7.72E + 09	1.20E + 07	1.36E + 05	Regulation AI-2	[28]	−6.47
Autoinducer 2- Modifying Protein LsrG		A0A2 × 3EW43	9.44E + 07	3.95E + 07	3.25E + 04	Regulation AI-2	[28]	−10.20
Autoinducer 2-Binding Protein LsrB		A0A422Z9T9	1.61E + 09	1.91E + 08	2.15E + 06	Transport AI-2	[27]	−6.43
S-Adenosylmethionine Synthase		A0AAE4MQH7	1.61E + 09	1.85E + 08	3.26E + 06	Production AI-2	[26]	−6.26
5’-Methylthioadenosine/S-Adenosylhomocysteine Nucleosidase		A0AAN1Y8 × 9	1.30E + 08	5.41E + 07	1.26E + 05	Production AI-2	[41]	−8.87
S-Ribosylhomocysteine Lyase (LuxS)		A0A2 × 3KKJ8	1.15E + 09	1.20E + 08	4.50E + 05	Production AI-2	[26]	−8.05
**PHAGE RECEPTORS**								
**DESCRIPTION**		**Accession no.**	**−10LgP**	**IAA**	**IAA+VAC7**	**Function**	**Ref.**	**log2FC**
LamB		A0A2 × 3CXE6	1.49E + 07	4.9E + 06	1.05E + 06	Receptor	[31]	−2.22
OmpK36		A0A2J4A8L8	1.77E + 09	1.47E + 08	3.29E + 07	Receptor	[32]	−2.15
OmpC		A0A346D4H7	1.7E + 09	1.4E + 08	2.9E + 07	Receptor	[31]	−2.33
FhuA		A0AB74GM38	1.24 + 09	9.10E + 06	5.33E + 05	Receptor	[33]	−2.99

Proteins associated with bacterial phage defense mechanisms were predominantly detected in the IAA-only condition and were not detected when phage infection occurred in the presence of IAA. These included GmrSD restriction endonuclease, a type IV modification-dependent restriction enzyme; bacteriophage control infection (BCI) (BCI), a prophage-associated infection control protein; and the mRNA interferase PemK, a toxin component of the PemIK type II toxin-antitoxin system. In contrast, abortive infection protein was detected in both conditions, although its abundance was reduced in the presence of phage.

### IAA Influences quorum sensing protein abundance

Several QS-related proteins were identified, with higher abundance observed in the IAA-only condition compared with the IAA-phage condition. These proteins included components involved in AI-2 biosynthesis (S-ribosylhomocysteine lyase (LuxS), S-adenosylmethionine synthase, and MTA/SAH nucleosidase), transport (LsrB), and signal regulation (LsrF and LsrG).

### Detection of phage receptors and phage proteins

Multiple bacterial proteins known to function as phage receptors, LamB, OmpC, OmpK36, and FhuA, were detected in both conditions but with lower abundance in presence of the phage (Table 2).

**Fig. 3:**
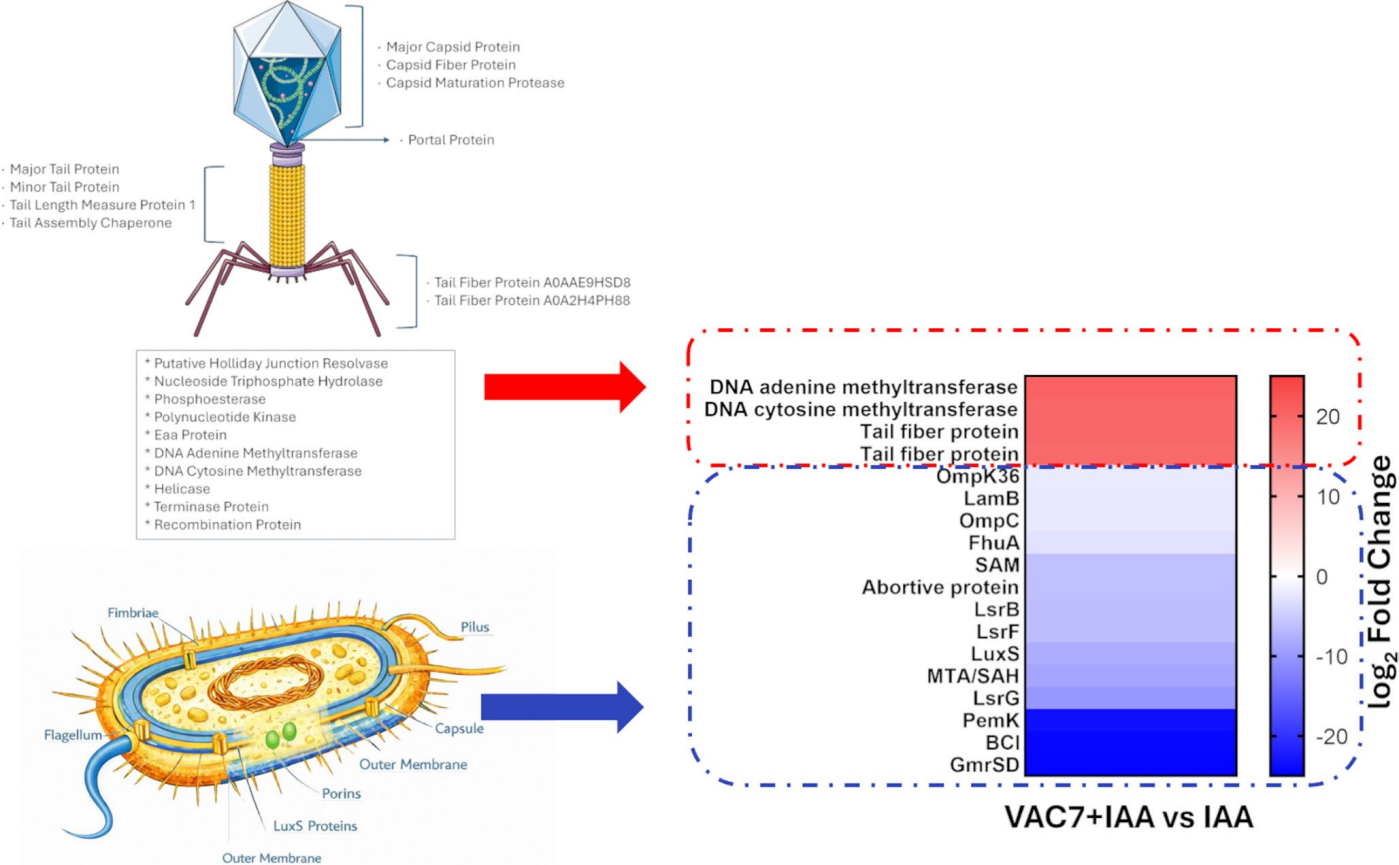
Representation of phage and bacterial proteins found in the proteomic analysis Heat map analysis. Illustrates relative changes in the abundance of specific proteins when comparing IAA + VAC7 condition respect IAA condition. Protein abundance changes are represented as log2-transformed FC values, where FC represents the ratio of protein abundance in the experimental condition relative to the control. Positive values (red) indicate increased protein abundance, neutral values with FC close to 0 are highlighted in white, and negative values (blue) indicate decreased abundance

In addition, 20 phage-encoded proteins were identified exclusively in the IAA plus phage condition (Fig. 3). These included structural proteins, replication-associated proteins, and proteins involved in counteracting bacterial defense systems. Among them were receptor-binding proteins (RBPs) (tail fiber proteins) and DNA adenine and cytosine methyltransferases, the last associated with inhibition of bacterial restriction-modification systems (Table 3).

**Table 3:** Phage proteins of proteomic analysis associated with anti-phage defense and Receptor Binding Proteins (RBP). The table includes Protein name; Accession number (database identifier); − 10LgP value, which represents the statistical confidence of protein identification; Abundance values associated with each condition; Biological function associated with each protein; Bibliographic references and log2-transformed FC values, that represent the ratio of protein abundance in IAA+VAC7 condition relative to the IAA condition. Positive values indicate increased protein abundance, whereas negative values indicate decreased abundance

ANTI-PHAGE DEFENSE and Receptor-Binding Proteins (RBPs)							
**DESCRIPTION**	**Accession no.**	**−10LgP**	**IAA**	**IAA+VAC7**	**Function**	**Ref.**	**log2FC**
Dna Adenine Methyltransferase	A0A0C5AFQ9	1.12E + 07	0.00E + 00	1.51E + 06	Anti-RM	[34]	20.52
Dna Cytosine Methyltransferase	A0AAE9HRL5	1.05E + 07	0.00E + 00	9.65E + 05	Anti-RM	[34]	19.87
Tail Fiber Protein	A0AAE9HSD8	1.34E + 06	0.00E + 00	6.90E + 05	RBPs	[42]	19.39
Tail Fiber Protein	A0A2H4PH88	9.82E + 06	0.00E + 00	8.93E + 05	RBPs	[42]	19.84

### Summary of proteomic changes

Heat map analysis of protein abundance changes revealed a general downregulation of bacterial phage defense proteins and QS-associated proteins in the IAA plus phage condition relative to IAA alone (Fig. 3). In contrast, phage-derived proteins exhibited high positive fold-change values, confirming enhanced phage activity and replication when IAA was present.

## Discussion

Despite the well-documented advantages of phage therapy, including high specificity, low toxicity, cost-effectiveness, and synergistic activity with antibiotics [4–6], the rapid emergence of bacterial resistance remains one of the principal barriers to its widespread clinical implementation. Bacteria possess a wide repertoire of phage defense mechanisms—such as restriction-modification systems, CRISPR-Cas immunity, abortive infection strategies, and QS-regulated responses—that enable them to rapidly adapt to phage pressure [9–13]. Consequently, the development of strategies capable of suppressing or bypassing these defenses is essential to enhance phage therapeutic efficacy.

In this context, the present study investigated whether indole-3-acetic acid (IAA), a naturally occurring auxin, could modulate QS signaling and thereby enhance phage infection in a multidrug-resistant *Klebsiella pneumoniae* clinical isolate. Our results demonstrate that exogenous IAA reduces expression of the QS regulator SdiA, alters QS gene and protein expression profiles, suppresses bacterial phage defense mechanisms, and ultimately enhances the efficacy of lytic phage VAC7 infection.

Previous studies have established that SdiA, an orphan LuxR-type receptor, plays a central role in regulating QS-dependent phenotypes in *K. pneumoniae*, including biofilm formation, fimbrial expression, and production of QS autoinducers [15]. Importantly, inhibition of SdiA has been associated with increased susceptibility to phage infection [17]. Consistent with these findings, our RT-qPCR results revealed a significant reduction in *sdiA* expression following exposure to subinhibitory concentrations of IAA, accompanied by increased expression of *luxS*, the gene responsible for AI-2 synthesis [26]. These data suggest a regulatory interaction in which SdiA negatively modulates AI-2–mediated QS signaling (Fig. 1), as previously proposed by Pacheco et al. [15], and demonstrate that IAA effectively disrupts this regulatory axis.

The QS-modulating activity of IAA was further supported by proteomic analyses, which revealed increased abundance of proteins involved in AI-2 biosynthesis, transport, and signal regulation under IAA-only conditions (Table 2). The presence of LuxS, LsrB, LsrF, and LsrG proteins indicates a fully active and tightly regulated AI-2 QS system [26–28]. In contrast, during phage infection in the presence of IAA, the abundance of these QS-related proteins was markedly reduced. This reduction likely reflects both the bacteriolytic effect of phage infection and the redirection of bacterial resources toward stress responses. Nevertheless, the initial QS modulation induced by IAA appears sufficient to impair the activation of key phage defense pathways.

Indeed, one of the most striking findings of this study was the pronounced reduction in bacterial phage defense proteins during phage infection in the presence of IAA (Table 2). Proteins associated with multiple defense systems—including the GmrSD type IV modification-dependent restriction endonuclease, the bacteriophage control infection (BCI) protein, and the mRNA interferase PemK—were detected only in the absence of phage and were not detected when phage infection occurred in the presence of IAA. This suggests that IAA, in association with its QS-modulator role, interferes with the basal or inducible expression of these defense mechanisms, thereby facilitating productive phage infection.

The absence of PemK toxin under IAA-phage conditions is particularly noteworthy. The PemIK type II toxin-antitoxin system has previously been shown by our group to induce reversible bacterial dormancy during phage infection, thereby limiting phage replication. In earlier proteomic analyses of the same *K. pneumoniae* ST16-OXA48 strain infected with phage VAC7, PemK expression was detected and associated with reduced phage propagation [29]. In contrast, the absence of PemK in the present study under IAA-phage conditions provide strong evidence that IAA suppresses this dormancy-based defense mechanism, allowing more efficient phage replication.

Similarly, the reduction of the BCI protein—previously described as a prophage-encoded defense system regulated by QS [30]—is consistent with the observed modulation of SdiA and reinforces the link between QS modulation and suppression of phage defenses. Although abortive phage infection resistance protein was detected in both conditions, its reduced abundance during phage infection in the presence of IAA suggests a diminished capacity to trigger programmed cell death, further favoring phage propagation.

IAA also appeared to influence bacterial susceptibility to phage infection by modulating the availability of phage receptors. Several outer membrane proteins known to function as phage receptors—LamB, OmpC [31], OmpK36 [32], and FhuA [33]—were detected under both experimental conditions, although they showed lower values in presence of VAC7. When a phage is present in the media, bacteria respond to the stress by reducing the presence of the receptors by different defensive strategies mediated in part by QS [34]. As with the QS proteins, in presence of IAA-phage the receptor protein values were reduced but not eliminated when compared with IAA-only, which led to an efficient infection. This is consistent with infection curve results and the phage-encoded proteins detected in the proteomic study.

The detection of multiple phage-encoded proteins exclusively under IAA-phage conditions provide direct evidence of active phage replication. These included structural proteins, RBPs, and enzymes involved in counteracting bacterial restriction–modification systems, such as DNA adenine and cytosine methyltransferases. These phage-encoded methyltransferases have been shown to protect phage DNA from host restriction enzymes, particularly type IV modification-dependent restriction systems [34]. The concomitant absence of host restriction proteins and presence of phage anti-restriction enzymes strongly supports the conclusion that IAA facilitates phage takeover of bacterial cellular machinery.

**Fig. 4:**
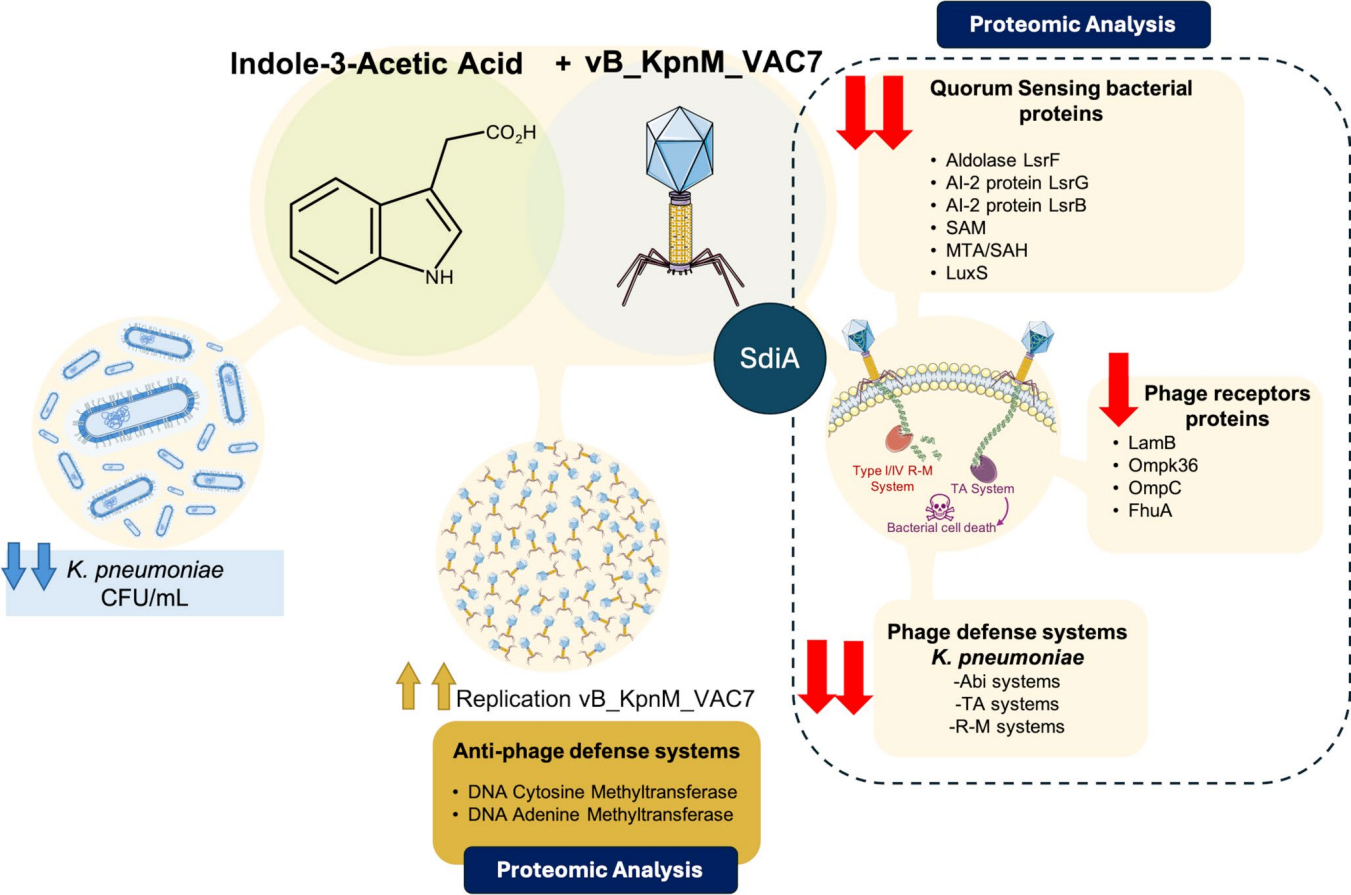
Schematic representation of the effect of Indole-3-acetic acid (IAA) on *K. pneumoniae* ST16-OXA48 in the presence of phage VAC7. IAA treatment reduces *K. pneumoniae* CFU/mL and is consistent with active phage replication. Additionally, leads to decreased phage defense systems (Abi, TA and R-M) in *K. pneumoniae* and modulates the expression of bacterial QS proteins (Aldolase LsrF, AI-2 proteins LsrG and LsrB, SAM, MTA/SAH, LuxS), phage receptor proteins (LamB, Ompk36, OmpC, FhuA) and anti-phage defense systems (DNA cytosine methyltransferase, DNA adenine methyltransferase), enhancing susceptibility to phage infection through the modulation of QS regulator SdiA

Taken together, the results of this study demonstrate that IAA-mediated modulation of the SdiA QS regulator leads to a coordinated suppression of bacterial phage defenses, enhancing phage infection and replication. These findings are summarized schematically in Fig. 4.

However, several aspects should be considered in future research, particularly the re-emergence of bacteria after 24 h. Proteomic assays indicate that, three hours after IAA application, bacterial defense mechanisms against phages are inhibited. Nevertheless, studies conducted in other Gram-negative bacteria, such as *Bradyrhizobium japonicum*, have demonstrated that the degradation kinetics of IAA must be taken into account, as they are proportional to the time elapsed after application. Specifically, at a concentration of 0.5 mM, approximately half of the compound had degraded after 8 h, and complete degradation was observed after 20 h [35]. These findings suggest a potential link between IAA degradation and the reactivation of bacterial defense mechanisms, ultimately leading to the re-emergence of the bacterial population. Another aspect has to do with the limitations of proteomic analysis itself, due to stochastic sampling effects, instrument detection thresholds, and technical limitations inherent to proteomic workflow, where preparation of protein sample is a critical initial step and directly affects the depth and accuracy of protein identification and quantification [36], absence of detection may not equate to biological absence. Additionally, extreme fold change values (e.g., log2FC = − 24) may represent methodological limitations as discussed above. Furthermore, the absence of a phage-only control is a limitation in this study and should be consider for a deeper understanding of the effect of IAA on phage infections. Finally, further investigation of the effects of IAA in *K. pneumoniae* biofilm activity is warranted. Recent studies in *Pseudomonas aeruginosa* have demonstrated that IAA can inhibit biofilm formation [37], and previous research in *K. pneumoniae* has shown that indole is capable of modulating biofilm development [38]. These findings support the need to evaluate the potential antibiofilm activity of IAA in this pathogen, particularly in biofilm-producing strains, as the *K. pneumoniae* ST16-OXA48 strain used in the present study is not a biofilm producer and is therefore not optimal for biofilm-related assays.

From a translational perspective, the use of natural compounds such as IAA represents a promising strategy to improve phage therapy efficacy. As a plant-derived auxin with demonstrated QS-modulating activity, IAA—or structurally related auxins—could be incorporated as adjuvants in phage-based treatments to reduce the emergence of phage resistance. More broadly, targeting QS pathways may represent one of the most effective approaches to sensitize MDR pathogens to phage infection. Further studies are warranted to evaluate the safety, pharmacodynamics, and in vivo efficacy of auxin-phage combinations and to explore their potential integration into future antimicrobial therapies.

## Supplementary Information

Below is the link to the electronic supplementary material.

Supplementary Material 1 (XLSX 260 KB)

Supplementary Material 2 (DOCX 52.0 KB)

## Data Availability

No datasets were generated or analysed during the current study.
